# Bacterial Distribution, Biogenic Amine Contents, and Functionalities of Traditionally Made Doenjang, a Long-Term Fermented Soybean Food, from Different Areas of Korea

**DOI:** 10.3390/microorganisms9071348

**Published:** 2021-06-22

**Authors:** Su-Ji Jeong, Myeong-Seon Ryu, Hee-Jong Yang, Xuan-Hao Wu, Do-Youn Jeong, Sun-Min Park

**Affiliations:** 1 Department of Research & Development, Microbial Institute for Fermentation Industry, Sunchang-Gun 56000, Korea; yo217@naver.com (S.-J.J.); rms6223@naver.com (M.-S.R.); godfiltss@naver.com (H.-J.Y.); 2 Department of Food and Nutrition, Obesity/Diabetes Research Center, Hoseo University, Asan 31499, Korea; niyani0@naver.com

**Keywords:** traditionally made doenjang, *Bacillus velezensis*, antioxidant activity, fibrinolytic activity, biogenic amine

## Abstract

Since doenjang quality depends on the bacterial composition, which ambient bacteria in the environment and production conditions influence, a complete understanding of the bacteria community in traditionally madetraditionally made doenjang (TMD) from different regions is needed. We aimed to investigate the bacteria composition and quality of TMD in the following areas: Chonbuk (CB), Chonnam (CN), Kyungsang (KS), Kangwon (KW), Chungchung (CC) provinces, and Jeju island (JJ) of Korea. Twenty-nine TMD samples from different regions were used to assess biogenic amine contents, bacteria composition using next-generation methods, and metabolic functions of the bacteria using Picrust2. *Bacillus* spp. were isolated, and their antioxidant and fibrinolytic activities were determined. Most TMD contained high amounts of beneficial bacteria (*Bacillus*, *Lactobacillus*, *Pediococcus* and *Weissella*). However, some KS samples contained harmful bacteria (*Cronobacter*, *Proteus* and *Acinetobacter*) and less beneficial *B. velezensis* bacteria. There was no similarity among the regional groups, and each TMD showed a different bacteria composition. Shannon index, α-diversity index, was lower in TMD from JJ and CB than the other areas, but there was no β-diversity among TMD from the six area groups. Picrust2 analysis revealed that the functional potential for arachidonic acid metabolism was lowest in JJ and CN, that for supporting insulin action was highest in KS and JJ, and that for carbohydrate digestion and absorption was lowest in CB and JJ among all groups (*p* < 0.05) according to the Kyoto Encyclopedia of Genes and Genomes Orthology. Histamine contents were lower in CN and CC, and tyramine contents did not differ significantly. *B. velezensis*, *B. subtilis*, *B. licheniformis*, *B. siamensis*, and *B. amyloliquefaciens* were isolated from TMD. None of the isolated *Bacillus* spp. contained the *B. cereus gene. B. subtilis* from CN had the highest fibrinolytic activity, and *B. velezensis* from CB had the highest antioxidant activity. In conclusion, TMD mainly contained various *Bacillus* spp., and the predominant one was *B. velezensis*, which had antioxidant and fibrinolytic activity regardless of the regional origin.

## 1. Introduction

Since Asians have consumed grains as the main staples of their diets, soybeans are a good source for complementing the limited amino acids of grains. However, dried soybeans require a long time to cook, and cooked soybeans can be rapidly spoiled before the introduction of refrigeration. Accordingly, in Asian countries, including Korea, China, Japan, India, and Indonesia, fermented soybeans have been consumed since the third century or earlier [1]. Fermented foods were reported to be consumed in Goguryeo Dynasty in AD 233–297, as described in Samgukji, a Korean history book written by Jinsu [1]. Samgukyusa, written by Il Yeon (AD 1206–1289), also described different types of fermented soybean foods with the functionality to promote wound healing [1].

In Korea, different types of fermented soybeans are collectively called “Jang” and are used for cooking [1]. Jang is mainly used as a sauce for soups and other side dishes in Asian countries. Different types of Jang are categorized according to the other added ingredients such as salt, grain, and red pepper, and fermentation periods such as short-term (2–5 days) and long-term (several months to years) [1]. Most types of Jang are pastes and are used as sauces, but soy sauce is a liquid fermented soy product.

Jang is traditionally prepared from meju, made by molding cooked soybeans into a rectangular box shape, drying outside, and fermenting at 23–25 °C for two weeks. Meju is naturally fermented with fungi such as *Aspergillus*, *Rhizopus*, *Mucor*, and *Penicillium* spp., and bacteria belonging to *Bacillus (B.)*, *Enterococcus*, and *Staphylococcus* spp. [2]. Bacterial compositions in Jang are dependent on production processes and conditions and the bacteria in the air and rice straw. Different provinces in Korea have somewhat different Jang production conditions due to somewhat different temperatures and salt contents according to regional preferences. Therefore, bacteria compositions and functionalities in Jang may be substantially different from the different provinces in Korea [2,3].

Meju is not used for cooking but needs a second fermentation in the salty water for 40–60 days, and then the salty water is separated to make kanjang [1]. The remaining soybean paste is kneaded, added with salt to make a 10–13% salt content, and fermented for an average of 6–12 months to make doenjang [4]. During the traditional natural fermentation of soybeans, the primary source of bacteria in traditionally made doenjang (TMD) is the air and rice straw, varying among the different provinces in Korea. The quality of TMD is influenced by environmental factors, including bacteria composition, fermentation temperature with seasonal variation and fermentation times, and salt contents. TMD quality is also affected by an intrinsic factor, pH, which is changed by the metabolites produced by the microorganisms [5]. The bacteria in some TMD change the composition of isoflavonoids and proteins to have better functionality, such as increased daidzein, genistein, equol, and smaller peptides, which may be quickly absorbed in the intestines [6]. According to the bacteria compositions, the types and contents of bioactive components of fermented soybeans differ in TMD. The bacteria composition plays a critical role in the quality of TMD.

Although some TMD has better taste and functionalities, including antioxidant and fibrinolytic activities, than commercially made doenjang [7], it is difficult to maintain a good sustainable quality of TMD, because the bacteria compositions are dependent on environments and management. TMD samples contain various bacteria in different areas of Korea, and their bacteria need to be identified to facilitate the production of better quality commercial doenjang. Commercial doenjang is made by inoculating cooked soybeans with starter microorganisms (koji). The taste and odor of the commercial doenjang depend on the microorganism in the koji. However, good microorganisms, especially bacteria, have not been developed for commercial doenjang for koji, even though commercial doenjang can be produced with consistent quality control. Koji is inoculated into cooked soybeans and salt and fermenting for about 60 days to become commercial doenjang [8]. Kim and Kwon [9] demonstrated that doenjang made with koji containing *Aspergillus oryzae*, *B.* *subtilis*, and *Zygosaccharomyces rouxii* has a better taste and lower biogenic amine contents than that made with *Aspergillus* only [9]. Currently, koji for commercial doenjang commonly contains *Aspergillus oryzae* and *Bacillus*. Although bacteria types and contents in koji play a key role in improving inoculated doenjang quality, few studies have been conducted for koji to make good-quality doenjang [9]. Research is needed to develop an optimal koji with various bacteria to improve the quality of commercial doenjang with good flavor and functionalities. The goal of the present study was to investigate the bacterial compositions and quality of TMD samples produced in different areas of Korea. Furthermore, we isolated and identified the bacteria species with no endotoxin and no biogenic amine-producing capacity and high antioxidant and fibrinolytic activities. The present study results would be primary data for developing optimized koji for high-quality commercial doenjang.

## 2. Materials and Methods

### 2.1. Sample Collection and Storage

TMD samples were collected from local markets in 9 different provinces, which covered all areas of Korea except Seoul, where no place for making TMD exists. The collected TMD included Chonbuk (5 samples), Chonnam (5 samples), Gyungkee (5 samples), Kyungbuk (3 samples), Kyungnam (3 samples), Kangwon (3 samples), Chungbuk (2 samples), and Chungnam (2 samples) province, and Jeju island (2 samples). We considered the area characteristics and sample size of TMD samples which were categorized into Chonbuk (CB), Chonnam (CN), Kyungsang (Kyungbuk + Kyungnam; KS), Kangwon (KW), Chungcheung provinces (Chungbuk + Chungnam, CC), and Jeju island (JJ). TMD from Gyungkee was categorized into Chungcheung and Kangwon based on the distance.

**Figure 1:**
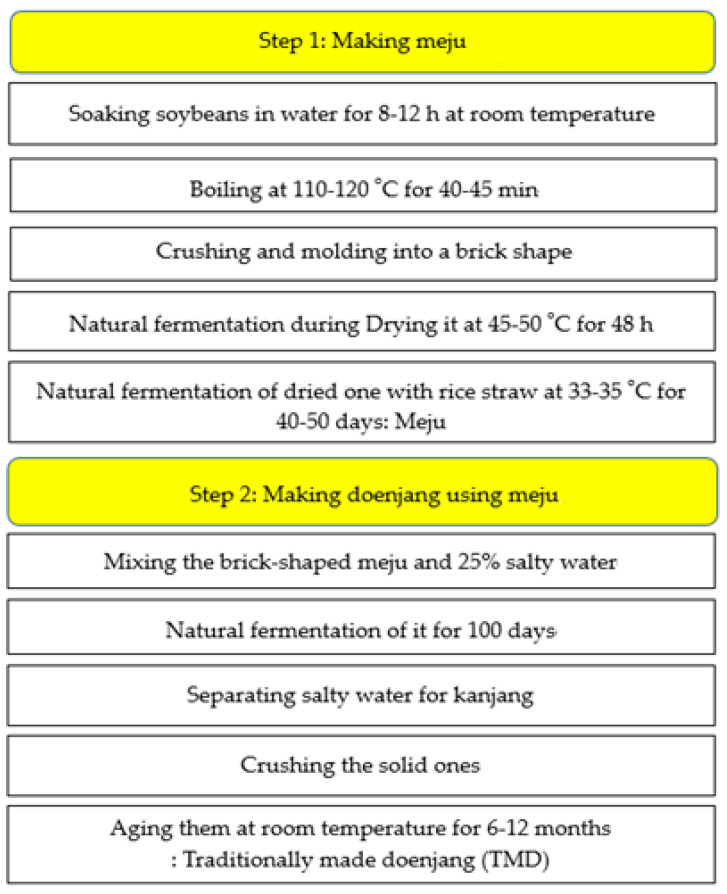
Processing steps of making traditionally made doenjang.

TMD was produced with a two-step fermentation method from crushed boiled soybeans (Figure 1): First, meju was made by molding boiled soybeans, naturally fermenting them at 45–50 °C for 48 h, and sequentially fermenting them 33–35 °C for 40–50 days with rice straw. The meju was then soaked in 25% salty water and naturally fermented for 100 days. The salty water was removed to make it into kanjang, and the remnants were naturally fermented for about one year, resulting in TMD. Twenty-nine TMD samples were collected in various areas of Korea. The collected samples were stored at 4 °C until further analysis.

### 2.2. DNA Extraction from the Collected Doenjang and Bacteria Analysis by the Next-Generation Sequencing (NGS) Method

DNA was extracted from the collected TMD samples using DNeasy PowerSoil Kit (Qiagen, Hilden, Germany). DNA quality and contents were checked with Qubit 1X dsDNA HS Assay kit (Invitrogen, Eugene, OR, USA), and DNA quality was confirmed by electrophoresis. The DNA was amplified with 16S amplicon primers targeting V3 and V4 regions using polymerase chain reaction (PCR). Each library was prepared using the PCR products according to the GS FLX plus library prep guide, and the DNA was amplified with 16S universal primers in the FastStart High Fidelity PCR System (Roche, Basel, Switzerland) described in a previous study [10]. Sequencing of bacterial DNA in the TMD samples utilized the Illumina MiSeq (Ann Arbor, MI, USA) standard operating procedure by a Genome Sequencer FLX plus (454 Life Sciences, Branford, CT, USA) in the Microbial Institute for Fermentation Industry (Soon Chang, Korea).

The 16S amplicon sequences were analyzed using Mothur v.1.36 (Ann Arbor, MI, USA). Using the Miseq SOP (Ann Arbor, MI, USA), the taxonomy and counts of the bacteria were evaluated in each doenjang sample. The sequences were aligned using Silva reference alignment v.12350 (Ann Arbor, MI, USA), and the taxonomy and bacteria counts of each taxonomic identity were determined. The relative bacteria number of each sample was calculated in the taxonomic assignments at the family, order, and genus levels after removing operational taxonomic units (OTUs) below 10,000 reads. Principle component analysis (PCA) was conducted using the R package with the OTU-abundance table converted to relative abundance. The α-diversity with Chao and Shannon indices was determined using the Mothur program.

### 2.3. Microbiota Function of the TMD Microbiota Predicted by PICRUSt2 Pipeline Analysis

Fasta and count table files produced by Mothur from TMD samples were used to make a biome file used to predict TMD bacteria’s metabolic activities using picrust2_pipeline.py provided (https://github.com/picrust/picrust2/wiki/Full-pipeline-script (accessed on 21 April 2021)). Predicted metabolic profiles for the Kyoto Encyclopedia of Genes and Genomes (KEGG) Orthology (KO) were mapped to various metabolism pathways that influence doenjang functionalities using the KEGG mapper (https://www.genome.jp/kegg/tool/map_pathway1.html (accessed on 31 April 2021)). An abundance of the mapped KO among different areas of TMD samples was analyzed with one-way ANOVA.

### 2.4. Isolation and Identification of Bacillus spp.

Since most *Bacillus* spp. in TMD are beneficial, they were isolated using nutrient agar media (Difco, NJ, USA) from each collected TMD sample. Each ground sample was put into sterile distilled water for 30 min, and each suspension was plated onto nutrient agar. Each plate, including the negative control (sterile distilled water without a sample), was incubated at 30 °C for 24 h. *Bacillus* spp. were screened for morphological differences, and they were isolated from the second incubation for verification.

*Bacillus* colonies were selected, and their bacterial species were identified after 16S rRNA sequencing and sequence alignment using NCBI BLAST. Bacterial DNA was extracted using a QIAamp DNA mini kit (QIAGEN, Hilden, Germany) according to the manufacturer’s protocol [11]. Amplification of the 16S rRNA gene was achieved using the PCR method according to the published protocol with some modifications [12]. Both strands of the purified PCR product with QIAquick PCR purification kit (Qiagen, Hilden, Germany) were sequenced with an ABI 310 automated sequencer according to manufacturers’ instructions (Perkin-Elmer, Foster City, CA, USA), using the proper PCR primers [13]. The PCR product sequence was compared with known 16S rRNA gene sequences in the NCBI GenBank database by multiple sequence alignment using the CLUSTAL W program (http://www.clustal.org/clustal2/ (accessed on 24 April 2021)).

### 2.5. Detection of B. Cereus Toxin Genes and Biogenic Amine Producing Genes

*B. cereus* is a Gram-positive and spore-forming food pathogen that causes food poisoning [14,15]. It is related to enterotoxins, including hemolysin BL (hbl), non-hemolytic enterotoxin (nhe), cytotoxin K (cytK), and enterotoxin FM (entFM) produced from B. cereus growth in the foods and small intestines. B. cereus existence in TMD samples was detected with the expression of cytK, nheA, entFM, bceT, hblC, and cer genes from genomic DNA isolated from bacteria identified by PCR [14,15]. Histidine decarboxylase (hdc) and tyrosine decarboxylase (tdc) gene expression indicating biogenic amine production was also measured from the genomic DNA with PCR. The PCR conditions and PCR primers for cytK, nheA, entFM, bceT, hblC, cer, hdc, and tdc were processed as described previously [14,15]. B. cereus was used for cytK, nheA, entFM, bceT, hblC, and cer gene expression, while the specific Bacillus with decarboxylase activity was used as the positive control for hdc and tdc expressions. A medium with no bacteria was considered as the negative control for all the PCR measurements. PCR products were identified with running electrophoresis in 2% agarose gel, and DNA bands for cytK, nheA, entFM, bceT, hblC, cer, hdc, and tdc expression were detected.

### 2.6. Characteristics and Functionalities of Isolated Bacillus spp. Were Evaluated

Biogenic amine production by isolated *Bacillus* during fermentation was determined as previously described [16]. Isolated *Bacillus* spp. were cultured in LB liquid media with 1000 ppm added Tyrosine (Sigma-Aldrich, St. Louis, MO, USA) and histidine (Sigma-Aldrich, St. Louis, MO, USA) in a shaking incubator at 37 °C for 24 h. After incubation, the supernatants were separated at 15,000 rpm for 30 min. Standards (histidine and tyramine) were made with 0.1–100 mg/L in 0.01 N HCl solution. An internal standard, 1,7-diaminoheptane (0.1 g/L, Sigma-Aldrich, St. Louis, MO, USA), was added into samples and standards (1:2, v:v) and then mixed with saturated Na_2_CO_3_ solution and 1% dansyl chloride (Sigma-Aldrich, St. Louis, MO, USA) to make derivatives. A total of 10% proline (Sigma-Aldrich, St. Louis, MO, USA) was added, and dansyl chloride was removed. Ethyl ether (Samchun, Seoul, Korea) was mixed with the solution for 3 min, and the supernatants were separated. The solvent was removed under nitrogen gas and the concentrates were dissolved into acetonitrile (Duksan, Seoul, Korea). The solution was filtered with a 0.45 μm syringe filter (Sartorius, Frankfurt, Germany) and then the contents of biogenic amines were measured [16].

Antioxidant and fibrinolytic activities were measured in each isolated bacteria from TMD samples. Antioxidant activities of isolated *Bacillus* spp. were measured with the capacity to remove free radicals by 2,2-diphenyl-1-picryl-hydrazyl (DPPH, Sigma-Aldrich, MO, USA) and superoxide dismutase (SOD)-like activity. After culturing isolated *Bacillus* spp. for 24 h, the culture media supernatants were separated after centrifugation at 13,000 rpm for 10 min, and 100 μM DPPH in ethanol was mixed to the samples (10:1) and incubated in a dark room for 30 min. The color changes were measured at 517 nm by UV/VIS spectrophotometer (SPECORD200, Analytik Jena, Jena, Germany). LB media (DifcoTM) was used for the negative control. DPPH free radical scavenging activity (%) was calculated with the equation: (1−Absorbance of DPPH solution) × 100.

SOD-like capacity was measured with a SOD kit (Sigma-Aldrich, St. Louis, MO, USA). In brief, samples were incubated with a working solution (10:1; v:v) and mixed with a working enzyme solution with the same sample volume. The mixture was incubated at 37 °C for 20 min, and optical density was measured at 450 nm.

The fibrinolytic activity of each isolated bacteria was measured by the fibrin plate method. Human fibrinogen was dissolved in 10 mM sodium phosphate buffer (pH 7.4) to a final concentration of 0.5%, followed by adding 100 unit/mL thrombin (Sigma-Aldrich, St. Louis, MO, USA) and 1% agarose (Bio-Rad, Hercules, CA, USA). The mixtures were solidified in a petri dish for 1 h at room temperature before use. Isolated *Bacillus* spp. were cultured in LB liquid media at 30 °C for 24 h, and each *Bacillus* spp. was separated after centrifuging the culture media at 13,000 rpm for 10 min. An aliquot of sterile saline containing each isolated bacteria (precipitates) was spotted on a fibrin plate followed by incubation at 37 °C for 24 h. The diameter of the cleared zone was measured [17].

### 2.7. Statistical Analysis

Statistical analysis was performed using SPSS version 20.0 (IBM Corp., Armonk, NY, USA). Results are presented as means ± standard deviations or frequency distributions. A one-way analysis of variance (ANOVA) was used to determine the significant differences among doenjang samples from different regions. Tukey’s test was conducted for multiple comparisons between groups. Statistical significance was accepted for *p*-values < 0.05.

## 3. Results

### 3.1. Bacterial Community Composition of TMD in Different Areas of Korea

**Figure 2:**
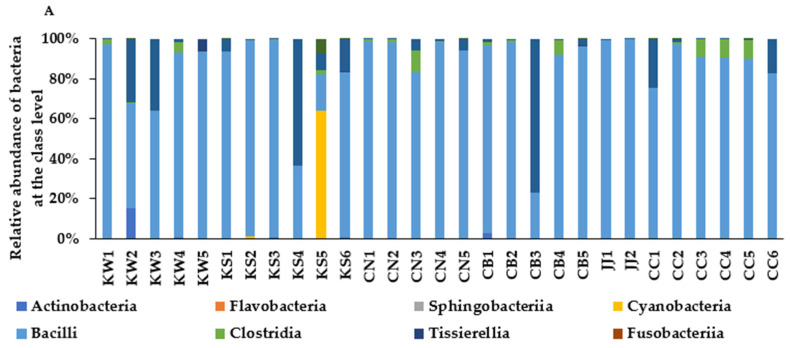

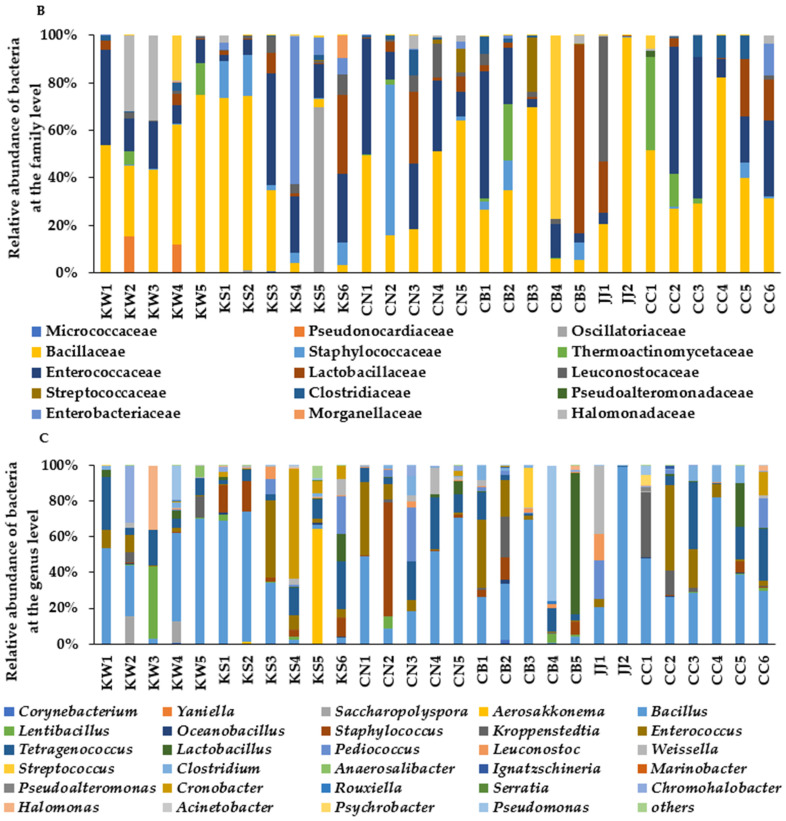

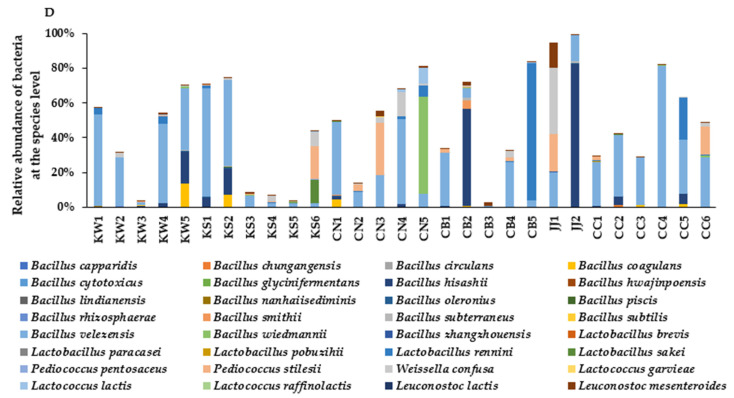
The bacterial composition of traditionally made doenjang (TMD). (**A**) Relative abundance at the class level of all bacteria. (**B**) Relative abundance at the family level of all bacteria. (**C**) Relative abundance at the genus level of all bacteria. (**D**) Percentage of *Bacillus* and *Lactobacillus* spp. at the species level. TMD samples from Chonbuk (CB), Chonnam (CN), Kyungsang (Kyungbuk + Kyungnam) (KS), Kangwon (KW), Chungcheung (Chungbuk + Chungnam) (CC), and Jeju island (JJ).

Bacteria communities of TMD showed that some *Bacillus* spp. were the primary bacteria at the class level in most areas in Korea. KS4, KS5, and CB3 contained higher amounts of gamaproteobacteria, cyanobacteria and gamaproteobacteria, respectively. Some TMD contained gamaproteobacteria, which are mostly harmful bacteria, but the relative contents of gamaproteobacteria were not significantly different among different regional groups (Figure 2A). Bacillaecae was the dominant bacteria in most TMD samples at the family level, whereas Lactobacillaceae was rich in some TMD (KS6, CN3, CB5, CC5, and CC6), which are beneficial bacteria (Figure 2B). However, *Enteroccoccacae* spp., the most harmful bacteria, were higher in KW1, KS3, KS6, CN1, CC2, and CC3. *B. velezensis* and *B. subtilis* were dominant in each TMD at the genus level, except KW3, KS4, KS5, KS6, CN2, CN3, CB4, and CB5 (Figure 2C).

At the species level, about 300 bacteria were identified in the NGS analysis of each TMD sample. Among them, the relative abundance of some *Bacillus, Lactobacillus*, *Pediococcus*, *leuconostoc*, and *Weissella* spp. were shown in each TMD sample (Figure 2D). The relative abundance of *B. velezensis* was highest in most TMD samples except KS6, CN5, CB2, and JJ2, although the bacterial composition was not different among the area groups. *B. hisashii*, *B. coagulans,* and *Pediococcus stilesii* were also abundantly present in some TMD (Figure 2D). Each TMD contained various bacteria in doenjang from different areas. Each TMD showed a different bacteria composition, and there was no similarity among the groups based on areas of origin.

### 3.2. α-Diversity and β-Diversity of TMD in Different Areas of Korea

**Figure 3:**
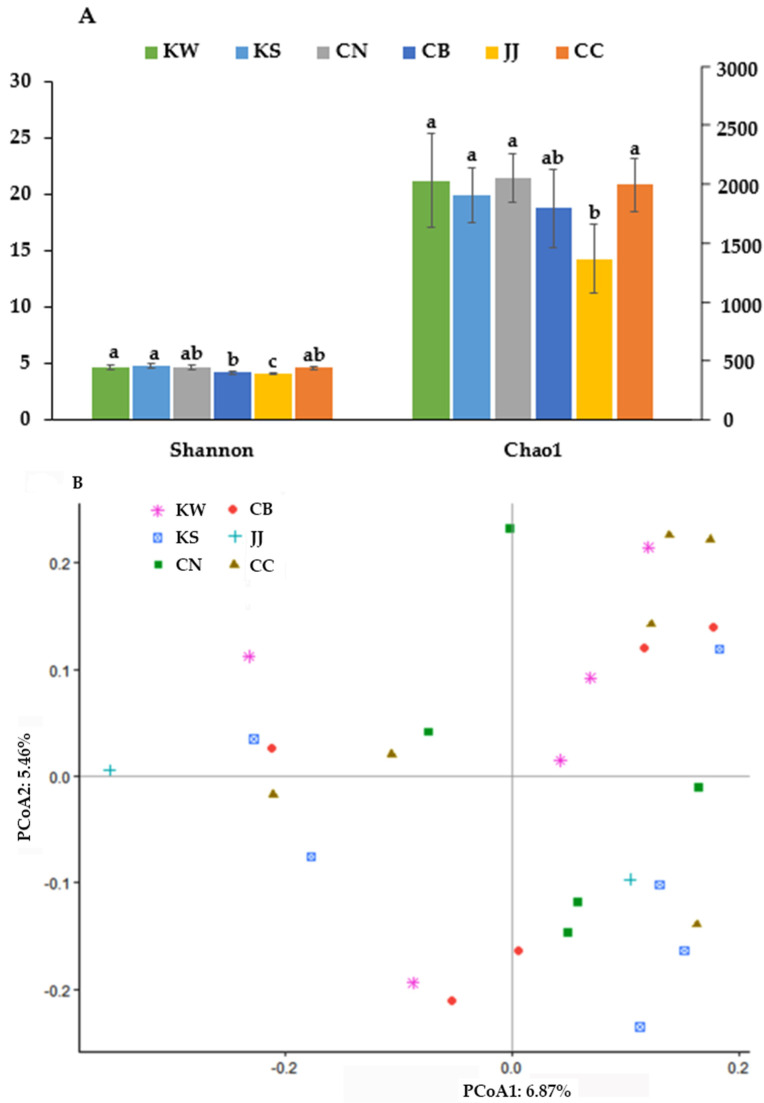
α-diversity and β-diversity of bacteria in traditionally made doenjang. (**A**) α-diversity, (**B**) β-diversity. A traditionally made doenjang (TMD) samples from Chonbuk (CB), Chonnam (CN), Kyungsang (Kyungbuk + Kyungnam; KS), Kangwon (KW), Chungcheung (Chungbuk + Chungnam; CC), and Jeju island (JJ). (**a**–**c**)—Different letters on the bars indicate significant differences among the groups by Tukey test (*p* < 0.05).

The bacterial compositions were varied in each area group, and there were no significant differences among the area groups. However, the species richness determined by Shannon and Chao1 index was significantly different among the groups (*p* < 0.05). The Shannon index representing bacteria richness was significantly lower in CB and JJ than in the other groups (Figure 3A). Thhe Chao1 index also showed similar patterns of Shannon index, but not significantly different (Figure 3A).

The PCoA1 and PCoA2 axis explained 6.87% and 5.46% of bacterial diversity in TMD samples. There was no significant separation among the regional groups of TMD samples (Figure 3B).

### 3.3. The Composition of Beneficial and Harmful Bacteria in Each TMD Samples

**Figure 4:**
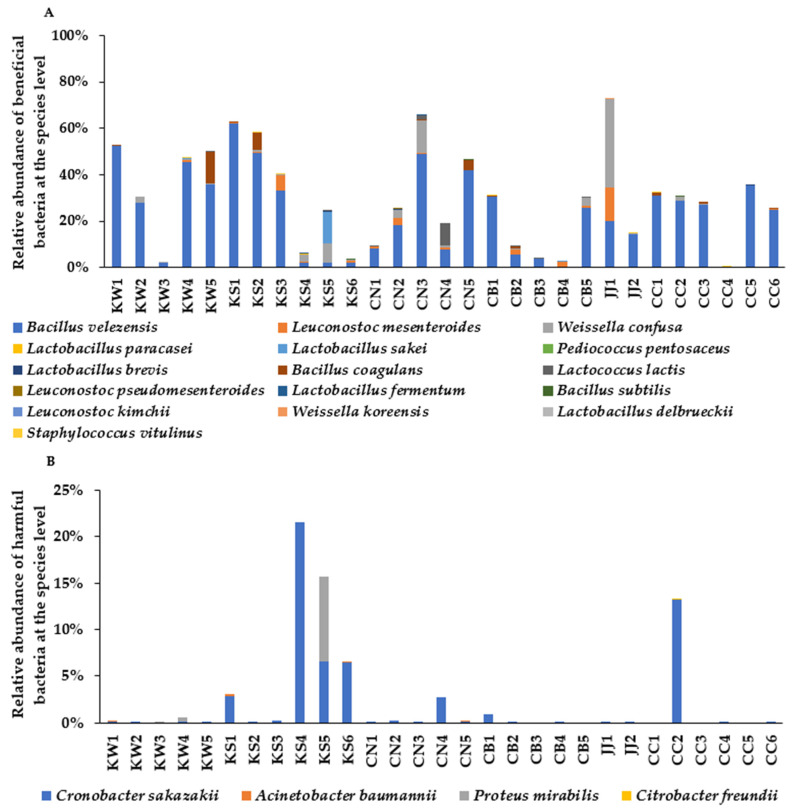
Beneficial and harmful bacteria in traditionally made doenjang (TMD). (**A**) The proportion of beneficial bacteria. (**B**) The proportion of harmful bacteria. TMD samples from Chonbuk (CB), Chonnam (CN), Kyungsang (Kyungbuk + Kyungnam; KS), Kangwon (KW), Chungcheung (Chungbuk + Chungnam; CC), and Jeju island (JJ).

Among the bacteria in TMD, the following bacteria were considered to be beneficial bacteria from previous literature: *B. velezensis*, *Leuconostoc mesenteroides*, *Weissella confuse*, *Lactobacillus paracasei*, *Lactobacillus sakei*, *Pediococcus pentosaceus*, *Lactobacillus brevis*, *B. coagulans*, *Lactococcus lactis*, *Leuconostoc pseudomesenteroides*, *Lactobacillus fermentum*, *B. subtilis*, *Leuconostoc kimchi*, *Weissella koreensis*, *Lactobacillus delbrueckii*, and *Staphylococcus vitulinus*. The contents of the bacterial species in each TMD are shown in Figure 4A. Among beneficial bacteria, the primary bacteria were *B. velezensis* in most doenjang samples. The second-ranked beneficial bacteria were *Weissella confuse* and *Leuconostoc mesenteroides*. Although the contents of beneficial bacteria seemed to be higher in the KW area than in other areas, it was not significantly different, since the beneficial bacteria contents in only one or two samples were different.

*Cronobacter sakazakii (Enterobacter sakazakii)*, *Acinetobacter baumannii*, *Proteus mirabilis*, and *Citrobacter* freundii were reported to be harmful to health [18,19,20,21]. KS4, KS5, KS6, and CC2 contained large amounts of *Cronobacter sakazakii*, the most common harmful bacteria (Figure 4B). KS5 also contained Proteus mirabilis. TMD in the KS area had higher harmful bacteria contents than KW, CN, CB, and JJ (*p* < 0.05).

### 3.4. Metabolic Activities of the Bacteria in Area Groups of TMD by PICRUSt2 Analysis

**Figure 5:**
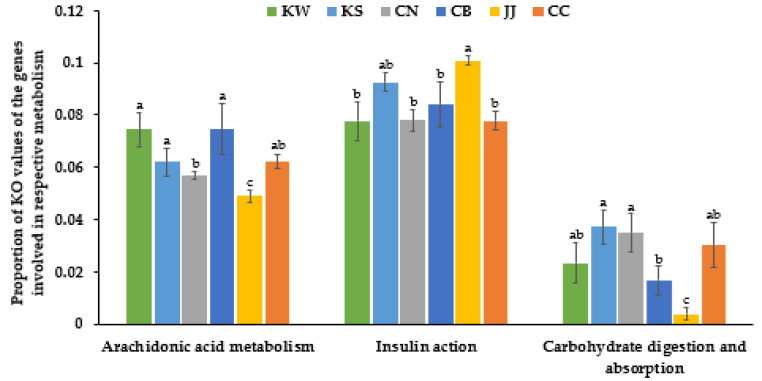
The relative abundance of the bacteria genes related to metabolic functions in traditionally made doenjang (TMD). Their relative abundance was calculated using the Kyoto Encyclopedia of Genes and Genomes Orthology (KO). TMD samples from Chonbuk (CB), Chonnam (CN), Kyungsang (Kyungbuk + Kyungnam; KS), Kangwon (KW), Chungcheung (Chungbuk + Chungnam; CC), and Jeju island (JJ). (**a**–**c**)—Different letters on the bars indicate significant differences between the groups by Tukey test (*p* < 0.05).

TMD samples among the different area groups show significant differences in the relative abundance of KO involved in arachidonic acid metabolism, pancreatic secretion, carbohydrate digestion and absorption, and insulin action, estimated by the genes expressed from bacteria compositions (*p* < 0.05; Figure 5). KW and CB exhibited a higher relative abundance of arachidonic acid metabolism-related species than CN, JJ, and CC, whereas KS and JJ had a higher relative abundance of insulin resistance-related genes than CN and CC (*p* < 0.05). The relative abundance of KEGG Orthologs involved in carbohydrate digestion and absorption was lower in CB and JJ groups than in KS and CN groups (*p* < 0.05; Figure 5).

### 3.5. Biogenic Amine Contents and Its Related Gene Expression in the Area Groups of TMD

Salt contents, which influence bacteria growth, were between 10.5–13.7% among TMD samples from the different areas. They were not significantly different among the different area groups, although the average salt contents tended to be lower in JJ and CC (Table 1).

Since common biogenic amines in TMD are histamine and tyramine, their contents in TMD samples were directly measured using HPLC analysis, and biogenic amine-producing gene expression was measured using PCR. Histamine contents were lower in the CN and CC groups than KW and KS groups, although each TMD had high variations (Table 1). However, tyramine contents tended to be lower in the KW and CB groups than the KS group, but it was not significantly different due to considerable variations of each TMD (Table 1).

In KEGG Orthology, the genes encoding enzymes responsible for the degradation of biogenic amines, histamine, and tyramine were not included. Thus, biogenic amine metabolism in different TMD cannot be determined by KEGG Orthology.

Biogenic amine-producing gene expressions in isolated *Bacillus* spp. were dependent on their strains. *B. subtilis* SRCM700686 contained biogenic amine production genes (hdc and tdc), but *B. subtilis* SRCM700659 did not express them in the KW group (Table 2). *B. velezensis* SRCM700669 and SRCM700754 included *hdc* gene, and *B. subtilis* SRCM700664 and SRCM700641 contained tdc genes in the KS group. The selected *Bacillus* in CN and CB did not include biogenic amine-producing genes (Table 2).

### 3.6. Antioxidant and Fibrinolytic Activities of Isolated Bacillus spp. of Each TMD from Different Areas

We isolated 3–4 *Bacillus* spp. from each TMD from different areas. The isolated *Bacillus* spp. included *B. velezensis*, *B. subtilis*, *B. licheniformis*, *B. siamensis*, *B. amyloliquefaciens*, and *B. sonorensis*. *B. velezensis* was the primary *Bacillus* spp. isolated from TMD. None of the *Bacillus* spp. isolated from TMD contained the *B. cereus* containing genes (Table 2).

Despite the same *Bacillus* spp., the characteristics were different, and it is due to different strains. Each isolated *Bacillus* spp. was identified by the strain numbers. We selected the *Bacillus* with better fibrinolytic and antioxidant activities among the isolated *Bacillus* spp., as shown in Table 2. The average radical removal percentage of all isolated *Bacillus* spp. in each area group was higher in JJ and CN than KS (Table 2). *B. atrophaeus* SRCM700770 from CN5, *B. velezensis* SRCM700762 from CC3, and *B. velezensis* SRCM700744 from KW4 removed over 20% of free radicals in TMD samples (Table 2). The SOD-like activity was measured in the isolated *Bacillus* spp. with >15% free radical removal capacity (Table 2). Although the SOD-like activity was not completely correlated with free radical removal capacity, their SOD capacity was over 30% (Table 2). *B. velezensis* SRCM700738 was highest at about 51%. *B. australimaris* in TMD from JJ, *B. atrophaeus* SRCM700770 from CN5, and *B. subtilis* SRCM700686 in TMD from KW2 had about 38% SOD activity (Table 2). Thus, antioxidant activity tended to be similar among TMD from the different areas.

Fibrinolytic activity exhibited a different pattern than antioxidant activity. KW, KS, and CN had higher average fibrinolytic activity than the other groups, and CC had the lowest fibrinolytic activity (Table 2). *B. subtilis* SRCM700689 from CN1 had similar fibrinolytic activity to the positive control (97.9%). *B. velezensis* SRCM700669 from KS2 had 84.4% of the fibrinolytic activity of the positive control.

Considering the antioxidant and fibrinolytic activities of TMD samples in different areas, *B. siamensis*, *B. subtilis*, and *B. velezensis* were selected in the KW group, and *B. velezensis* SRCM700744 had the highest fibrinolytic activity among the selected *Bacillus* spp. In KS, *B. velezensis*, *B. licheniformis*, and *B. subtilis* were isolated, and *B. subtilis* SRCM700752 exhibited higher antioxidant and fibrinolytic activities than the others. In CN, *B. siamensis*, *B. velezensis*, and *B. atrophaeu* were isolated, and *B. velezensiss* SRCM700717 showed higher antioxidant and fibrinolytic activities than the other strains. In CB, *B. amyloliquefaciens*, *B. sonorensis*, and *B. velezensis* were separated, and all of them had higher fibrinolytic activity than 53 mm. In CC, *B. siamensis* and *B. velezensis* were isolated, and *B. velezensis* SRCM700754 had higher antioxidant and fibrinolytic activities than the other strains. These results indicated that *Bacillus* spp. compositions in TMD have the most significant influence on the functional quality of TMD.

## 4. Discussion

TMD is a long-term naturally fermented soybean food with 10–15% salts and rich in diverse microorganisms, mainly *Bacillus* spp. However, it is challenging to control harmful bacteria and toxins such as *B.* cereus and aflatoxin to make good TMD. About 40 years ago, doenjang was made in individual homes, but inoculated doenjang or TMD is currently purchased for cooking by most people due to lifestyle changes. The bacteria in TMD need to be identified and isolated to make koji for inoculating soybeans when making commercial doenjang. TMD has a better taste, less biogenic amines, and less harmful bacteria than inoculated doenjang [22]. Therefore, beneficial microorganisms from high-quality TDM need to be isolated. The present study identified the bacterial composition of 29 TMD from different provinces in Korea by the NGS method, and their biogenic amine contents were determined. Moreover, some *Bacillus* spp. were isolated from TMD, and their functionality, including antioxidant and fibrinolytic activity, and genes linked to biogenic amine production and *B. cereus* identification, were measured. The present study did not investigate the fungi and yeast in TMD, since bacteria contribute most of the bioactivities in TMD. Previous studies demonstrated that the most common bacteria in TMD are *B. amyloliquefaciens* and *Tetragenococcus halophilus*, whereas, among fungi, doenjang does not contain *Aspergillus* but contains *Candida versatilis*, *Mucor racemosus*, and *Penicillium expansum* [23]. We expected that the TMD from the different areas might have different bacteria compositions due to different environmental bacteria. However, each TMD has a peculiar bacteria composition: TMD samples from the same or different provinces exhibited high variations of bacteria. These results indicated that the bacterial composition of each TMD was different, and it depends on the environmental conditions during the over one year of fermentation outdoors.

Various factors, such as bacteria in rice straw, fermentation time, and management of fermentation conditions, influence the bacterial composition of TMD. It changes metabolites of soybeans, and pH in TMD is normally about 5.3 and varies between 4.9–6.2. The pH discrepancy in TMD is related to the contents of *Lactobacillus* spp., yeast, and organic acids according to the fermentation periods [24]. In the present study, although pH was not measured in TMD, *Lactobacillus* spp. contents were calculated. The relative abundance of *Lactobacillus* spp. ranged from about 1–2% regardless of the areas, but one of KS, CN, and CB was much higher than other TMD. Our previous study showed that the rice straw used for meju fermentation contains 71% Proteobacteria, 20.6% Actinobacteria, 4.2% Bacteroidetes, and 1.3% Firmicutes according to pyrosequencing analysis [25]. Meju also contains 77% *Bacillus,* 14.5% *Enterococcus,* 3.65%, *Pediococcus,* and 1.8% *Clostridium,* whereas the long-term fermented TMD contains 92.2% *Bacillus. B. subtilis*, *B. licheniformis, B. amyloliquefacience,* and *B. atrophaeus* are predominant bacteria in long-term fermented doenjang. The longer the fermentation time of TMD, the higher *Bacillus* spp. contents, especially *B. amyloliquefacience* and *B.* *velezensis* [25]. The predominant bacteria in TMD produce metabolites that have anti-bacterial activity against harmful bacteria, including *B. cereus* and *Staphylococcus,* and *Enterococcus* spp. The beneficial bacteria in TMD can reduce harmful bacteria growth. The present study also demonstrated that the predominant bacteria is *Bacillus* spp. in all TMD, and at the species level, *Bacillus velezensis* was the highest one, and *Leuconostoc mesenteroides* was the second most prevalent bacteria in TMD. *Lactococcus lactis, Weissella confuse*, and *Lactobacillus sakei* are also present in high amounts in some TMD. However, there were no specific characteristics of the bacterial community according to the region of origin of the TMD. Three-month fermented doenjang with different salt concentrations has been demonstrated to have *Enterococci* as predominant bacteria [26]. Therefore, fermentation periods and salt contents influence the bacteria community.

Biogenic amines are collectively referred to as compounds containing one or more amine groups. They are synthesized by microbial decarboxylation of amino acids in foods, especially fermented foods. Biogenic amines act as neurotransmitters to regulate vascular function. However, they can induce allergy and be converted into cancerous compounds such as N-nitrosamine. Biogenic amines are potentially harmful substances of doenjang. Several biogenic amines in doenjang have been reported; for example, tryptamine, β-phenylethylamine, putrescine, cadaverine, histamine, tyramine, spermidine, and spermine [22]. Biogenic amine contents are influenced by bacterial composition in doenjang. Some TMD contains different biogenic amines, especially tyramine, spermidine, spermine, and histidine, which are higher in some TMD [27]. The present study demonstrated that histamine contents were lower in CN and CC, and tyramine contents were not significantly different. Although salt contents may be related to biogenic amine production, there were no significant differences in TMD salt contents from different areas. Doenjang having lower salt contents have higher biogenic amine contents [28]. However, in the present study, CN and CC had about 10.5% and 11.5%, which was not as high in salt content as other doenjang samples (about 15%). In recent years, the Korean government has released public health messages encouraging people to eat less salt for health reasons, and the salt contents in Jang have been reduced. Shukla et al. has reported that doenjang made with the inoculation of fungi with *B. subtilis* TKSP 24 contains negligible biogenic amine formation [29]. The present study also showed that some *B. velezensis, B. subtilis*, and *B. amyloliquefaciens* did not include biogenic amine (tyramine and histamine)-producing genes involved in allergic reactions. *Bacillus* spp. without biogenic amine-producing genes can be candidates for making inoculated doenjang without producing biogenic amines.

*B. cereus* is a major type of toxic bacteria detected in some doenjang. However, in the present study, *B. cereus* genes (cytK, nheA, entFM, bceT, hblC, and cer) were not detected in the 29 types of TMD selected from different areas. In addition, some *Bacillus* spp. like *B. tequilensis* have been reported to inhibit *B. cereus* growth [30]. When beneficial *Bacillus* spp. are predominant in TMD, harmful *Bacillus* spp. like *Bacillus cereus* can be suppressed [30]. In the present study, KS4, KS5, KS6, and CC2 contained the foodborne pathogen *Cronobacter sakazakii*, which can cause bacteremia and sepsis, cerebrospinal, and peritoneal fluid accumulation, and brain abscesses [31]. *Cronobacter sakazakii* infection increases mortality rates up to 80%, especially in infants and neonates [31]. Therefore, TMD needs to be manufactured in ways that prevent harmful bacteria growth and encourage high contents of beneficial bacteria.

TMD has been reported to have various functionalities such as anti-cancer, immune modulation, antioxidant, and anti-fibrinolytic activity, angiotensin-converting enzyme, and tyrosinase inhibitory activity [32,33]. These activities are associated with the soybean composition, bacteria, and metabolites. These functions are involved in the genes of the bacteria. Using KEGG Orthology, the molecular function of genes in the bacteria showed statistical differences of arachidonic acid metabolism, insulin resistance, and carbohydrate digestion and absorption among the TMD from different areas. These results indicated that the bacteria in TMD changed this metabolism in soybean fermentation. After TMD intake, this metabolism can be altered in humans. Further studies are needed to study the effects of TMD on molecular functions in animals and humans.

In the present study, the predominant bacteria were isolated from TMD from different areas in Korea, and their antioxidant and anti-fibrinolytic activity were determined. The present study demonstrated that the most predominant *Bacillus* spp. in various TMD was *B. velezensis* (25–63%). *B. velezensis* is known to suppress the growth of pathogens by the biosynthesis of secondary metabolites, including cyclic lipopeptides and polyketides [34]. It also exhibits antifungal activity [34]. Therefore, the predominance of *B. velezensis* in TMD has the potential to promote the suppression of harmful microbiota in TMD. However, some TMD in the KS area contained low *B. velezensis* counts (1.9–3.4%), and they had relatively high *Cronobacter sakazakii,* harmful bacteria, suggesting that high *Bacillus velezensis* contents inhibit the growth of *Cronobacter sakazakii*.

*Bacillus* spp. in the short-term fermented soy food, chungkookjang, are well-known to have fibrinolytic and antioxidant activity [35]. The dominant and essential bacteria in TMD are *B.* *velezensis, B. subtilis, B. licheniformis, B. siamensis,* and *B. amyloliquefaciens*, some of which are also *dominant Bacillus* spp. in chungkookjang [36]. The chungkookjang made with *B. amyloliquefaciens, B. subtilis,* and *B. licheniformis* had various functions such as fibrinolytic and antioxidant activities [35,37]. The functionality of *Bacillus* spp. depends on the species and specific strains of *Bacillus* spp., which need to be identified to make koji for doenjang. The present study demonstrated that most isolated *Bacillus* spp. had high fibrinolytic activity: *B. subtilis* SRCM700689 from CN1 had the highest fibrinolytic activity, similar to the positive control. However, only some isolated *Bacillus* spp. had antioxidant activity: *B. velezensis* SRCM700738 and *B. atrophaeus* SRCM700770 had about 50% SOD-like activity. Three-month fermented doenjang has been demonstrated to have antioxidant activity with lower salt contents (about 8%) [26], but the study did not show which components of doenjang have antioxidant activity. Previous studies have revealed that *B. velezensis* and subtilis have antioxidant activities [38,39]. Therefore, different types of TMD have different antioxidant and fibrinolytic activities due to different *Bacillus* spp. and their secondary metabolites.

## 5. Conclusions

Most TMD samples contained high beneficial bacteria (*Bacillus*, *Lactobacillus*, *Pediococcus*, and *Weissella* spp.). However, some KS contained harmful bacteria (*Cronobacter*, *Proteus*, and *Acinetobacter* spp.). TMD containing the high contents of *Cronobacter* tended to be lower in *B.* *velezensis* beneficial bacteria. There was no *B. cereus* in any TMD. However, the bacterial community of TMD was not dependent upon the region where it was produced due to the variabilities of bacteria composition in TMD samples of the same area. Biogenic amine contents in TMD depended on containing *Bacillus* spp., which expressed the biogenic amine-producing genes. Biogenic amine contents were lower in CN and CC, which contained *Bacillus* spp. without expressing the biogenic producing gene. *B. subtilis* from CN had the highest fibrinolytic activity, and *B. velezensis* from CB had the highest antioxidant activity. TMD mainly contained *Bacillus* spp., and the predominant *B. velezensis* *and B. subtilis* had antioxidant and fibrinolytic activity regardless of the region of origin. Furthermore, the functionalities of TMD were dependent on bacteria strains. The inoculated doenjang made with koji containing the specific strains of *B. velezensis* and *B. subtilis* would be protective against harmful bacterial growth and contribute to the antioxidants and fibrinolytic activities.

## Figures and Tables

**Table 1:** Histamine, tyramine, and salt contents in traditionally made doenjang (TMD) samples from different areas of Korea.

	KW (*n* = 5)	KS (*n* = 6)	CN (*n* = 5)	CB (*n* = 5)	JJ (*n* = 2)	CC (*n* = 6)
NaCl (%)	11.5 ± 0.6	11.7 ± 2.6	11.5 ± 1.4	13.7 ± 2.8	10.8 ± 0.2	10.5 ± 4.1
Histamine (mg/kg TMD)	574 ± 116 ^a^	574 ± 137 ^a^	266 ± 61 ^b^	334 ± 74 ^a,b^	434 ± 158 ^a,b^	263 ± 88 ^b^
Tyramine (mg/kg TMD)	317 ± 79	709 ± 263	461 ± 148	403 ± 112	584 ± 141	540 ± 146

CB, Chonbuk; CN, Chonnam, KS, Kyungsang (Kyungbuk + Kyungnam), KW, Kangwon, CC, Chungcheung (Chungbuk + Chungnam), JJ, Jeju island. ^a,b^ Different letters on the bars indicate significant differences between the groups by Tukey test (*p* < 0.05).

**Table 2:** Characteristics of isolated *Bacillus* spp. from each traditionally made doenjang.

Sample	SRCM No.	16S ID	Biogenic Amine Producing Gene		Radical Removing Capacity (DPPH %) ^a^	SOD-Like Activity (%) ^b^	Fibrinolytic Activity (%) ^c^
			Hdc	Tdc			
KW1	SRCM700667	*B. siamensis*	−	−	5.69 ± 1.7	−	38.4 ± 2.23
KW2	SRCM700686	*B. subtilis*	+	+	17.9 ± 0.73	38.5 ± 4.06	45.2 ± 0.71
KW3	SRCM700659	*B. subtilis*	−	−	13.6 ± 0.57	−	41.5 ± 0.91
KW4	SRCM700744	*B. velezensis*	−	−	22.8 ± 1.44	7.30 ± 1.88	41.2 ± 3.08
KW5	SRCM700721	*B. velezensis*	−	−	6.45 ± 0.52	−	71.0 ± 4.26
KS1	SRCM700704	*B. licheniformis*	−	−	19.3 ± 0.23	23.7 ± 1.04	53.6 ± 2.44
KS2	SRCM700669	*B. velezensis*	+	−	5.97 ± 1.13	−	84.4 ± 0.74
KS3	SRCM700752	*B. subtilis*	−	−	17.0 ± 0.38	32.4 ± 5.73	71.6 ± 1.29
KS4	SRCM700623	*B. velezensis*	−	−	8.38 ± 2.05	−	46.0 ± 0.58
KS5	SRCM700664	*B. subtilis*	−	+	4.19 ± 1.22	−	39.6 ± 2.17
KS6	SRCM700699	*B. velezensis*	−	−	10.7 ± 1.18	−	78.2 ± 4.4
CN1	SRCM700689	*B. subtilis*	−	−	10.2 ± 0.33	−	97.9 ± 1.22
CN2	SRCM700612	*B. velezensis*	−	−	13.2 ± 0.47	−	50.6 ± 0.78
CN3	SRCM700634	*B. siamensis*	−	−	10.3 ± 0.32	−	40.7 ± 0.10
CN4	SRCM700717	*B. velezensis*	−	−	15.5 ± 1.66	30.4 ± 6.25	69.4 ± 0.37
CN5	SRCM700770	*B. atrophaeus*	−	−	25.1 ± 1.36	38.0 ± 0.00	46.7 ± 2.91
CB1	SRCM700738	*B. velezensis*	−	−	20.1 ± 0.37	51.2 ± 6.67	36.6 ± 0.44
CB2	SRCM700614	*B. velezensis*	−	−	7.99 ± 0.71	0	60.4 ± 1.79
CB3	SRCM700629	*B. velezensis*	−	−	9.88 ± 1.04	−	47.6 ± 1.39
CB4	SRCM700706	*B. amyloliquefaciens*	−	−	19.4 ± 0.71	32.9 ± 4.27	49.9 ± 1.05
CB5	SRCM700748	*B. sonorensis*	−	−	14.7 ± 1.14	−	52.7 ± 6.02
JJ1	SRCM700650	*B. velezensis*	−	+	11.2 ± 0.56	−	35.2 ± 3.28
JJ2	SRCM700764	*B. australimaris*	−	+	17.9 ± 1.07	39.8 ± 6.85	49.0 ± 1.83
CC1	SRCM700641	*B. subtilis*	−	+	14.4 ± 1.03	−	47.8 ± 0.91
CC2	SRCM700645	*B. velezensis*	−	−	9.43 ± 1.2	−	58.9 ± 0.27
CC2	SRCM700655	*B. velezensis*	−	−	11.8 ± 1.35	−	18.6 ± 26.3
CC3	SRCM700762	*Bacillus velezensis*	−	−	24.7 ± 1.36	32.7 ± 1.33	47.1 ± 0.24
CC4	SRCM700734	*Bacillus siamensis*	−	−	12.6 ± 0.53	−	40.8 ± 3.82
CC6	SRCM700754	*Bacillus velezensis*	+	−	16.2 ± 0	7.15 ± 1.67	63.4 ± 1.18

Values represented mean ± SD. ^a^ DPPH free radical scavenging activity (%) = (1 − Absorbance of DPPH solution) × 100 Measure SOD activity when the samples have over 15% DPPH. ^b,c^ Relative activity based on the positive-control.

## Data Availability

Data are available from the corresponding authors on reasonable request with a reasonable reason.
